# Modification of translationally controlled tumor protein-derived protein transduction domain for improved intranasal delivery of insulin

**DOI:** 10.1080/10717544.2018.1464081

**Published:** 2018-04-24

**Authors:** Hae-Duck Bae, Joohyun Lee, Kyu-Yeon Jun, Youngjoo Kwon, Kyunglim Lee

**Affiliations:** Graduate School of Pharmaceutical Sciences, College of Pharmacy, Ewha Womans University, Seoul, Korea

**Keywords:** Drug delivery, insulin, intranasal absorption, protein transduction domain, translationally controlled tumor protein

## Abstract

Carrier peptides, termed protein transduction domains (PTDs), serve as provide promising vehicles for intranasal delivery of macromolecular drugs. A mutant PTD derived from human translationally controlled tumor protein (TCTP-PTD 13, MIIFRALISHKK) was reported to provide enhanced intranasal delivery of insulin. In this study, we tested whether its efficiency could be further improved by replacing amino acids in TCTP-PTD 13 or changing the amino acids in the carrier peptides from the l- to the d-form. We assessed the pharmacokinetics of PTD-mediated transmucosal delivery of insulin in normal rats and the activity of insulin in alloxan-induced diabetic rats. The safety/toxicity profile of the carrier peptides was evaluated based on the release of lactate dehydrogenase (LDH) in nasal wash fluid, body weight changes, and several biochemical parameters. Pharmacokinetic and pharmacodynamic studies showed that the l-form of a double substitution A6L, I8A (MIIFRLLASHKK), designated as l-TCTP-PTD 13M2 was the most effective carrier for intranasal insulin delivery. The relative bioavailability of insulin co-administered intranasally with l-TCTP-PTD 13M2 was 37.1% of the value obtained by the subcutaneous route, which was 1.68-fold higher than for insulin co-administered with l-TCTP-PTD 13. Moreover, co-administration of insulin plus l-TCTP-PTD 13M2 reduced blood glucose levels compared to levels in diabetic rats treated with insulin plus l-TCTP-PTD 13. There was no evidence of toxicity. These results suggest that the newly designed PTD is a useful carrier peptide for the intranasal delivery of drugs or biomolecules.

## Introduction

Intranasal administration is often a preferred approach to drug delivery as it offers a needle-free route and allows rapid absorption of drugs into the circulation and avoids hepatic first-pass metabolism. However, because intranasal absorption of therapeutic macromolecules (e.g. peptides and proteins) is limited by their large size, their hydrophilicity, poor permeation across the nasal epithelium, and their susceptibility to rapid enzymatic degradation in the nasal cavity, the bioavailability of peptide and protein drugs such as insulin administered nasally, is generally low (McMartin et al., 1987; Arora et al., 2002). Therefore, different approaches for enhancing the intranasal absorption of poorly absorbable drugs through the nasal mucosa have been investigated, including the use of permeation enhancers, enzyme inhibitors and cationized polymers (Merkus et al., 1993; Marttin et al., 1995; Duan & Mao, 2010).

Protein transduction domains (PTDs) are short peptides that not only penetrate cell membranes but also serve as carriers for the *in vitro* and *in vivo* cellular delivery of cargo molecules including plasmid DNA, small interfering RNA, proteins and nanoparticles (Jarver & Langel, 2004; El-Andaloussi et al., 2005; Guo et al., 2016). To date, three strategies have been described for cargo delivery using PTDs: fusion of the cargo to a PTD, covalent conjugation of the cargo to a PTD, and simple mixing of the cargo with a PTD (Nagahara et al., 1998; Morris et al., 2001, 2008; Zatsepin et al., 2005). The first two approaches require the expression of PTD-fused cargo and chemical cross-linking between the cargo and PTD, respectively, while simply mixing a PTD with a cargo requires complexation via electrostatic, hydrophobic or other interaction (Gros et al., 2006). Such strategies have been used to enhance nasal and intestinal absorption of therapeutic peptides (Liang & Yang, 2005; Khafagy et al., 2009; Sakuma et al., 2010; Kristensen et al., 2015b).

We previously reported that the human translationally controlled tumor protein (TCTP) contains a PTD at its N-terminus (TCTP-PTD) with the amino acid sequence MIIYRDLISH (residues 1–10) (Kim et al., 2011a). To improve the penetration of peptides through the cell membrane, we designed variant peptides modified from TCTP-PTD. Several variants of TCTP-PTD significantly enhanced the transduction compared with that of the wild-type TCTP-PTD (Kim et al., 2011b). Among them, TCTP-PTD 13 consisting of l-amino acids (1-MIIFRALISHKK-12), enhanced nasal absorption compared to administration of insulin alone (Bae & Lee, 2013).

In the present study, we designed novel TCTP-PTD analogs to further improve nasal insulin absorption and evaluated their efficacy in normal rats. We also assessed the hypoglycemic effect of insulin nasally co-administered with PTD in a rat model of alloxan-induced diabetes and investigated their toxicity of TCTP-PTD analogs reflected by lactate dehydrogenase (LDH) levels in nasal wash fluid, body weight changes, and several biochemical parameters.

## Materials and methods

### Materials

Human recombinant insulin (27.5 IU/mg), sodium taurodeoxycholate and alloxan monohydrate were purchased from Sigma-Aldrich (St. Louis, MO). TCTP-PTD analogs were synthesized by Peptron Co., Ltd., (Daejeon, Korea). All peptides were acetylated at the N-terminus while their C-termini were protected by amidation. N-terminal fluorescein isothiocyanate (FITC)-labeled peptides were also synthesized and used to evaluate the cellular uptake efficiency. The purity of the peptides assessed by high-performance liquid chromatography was >95%. All the chemicals used in this study were of analytical grade.

### Animals

Male Wistar rats and ICR mice (5-week old) used in these studies were purchased from the Young Bio Co., Ltd. (Seoungnam, Korea). They were housed under controlled humidity and temperature, on a 12-h light/dark cycle with free access to food and water. Rats weighing 160–200 g were fasted overnight with continued access to water before the experiments. All animal experiments were approved by Ewha Womans University’s Institutional Animal Care and Use Committee (approval ID: 14-095).

### Cellular uptake assay

Cellular uptake assays were carried out to assess the membrane permeability of the new peptides, as previously described (Bae et al., 2016). Briefly, BEAS-2B human bronchial epithelial cells (American Type Culture Collection [ATCC], Manassas, VA) were maintained in bronchial epithelial growth medium (BEGM; Lonza, Walkersville, MD) and 2 × 10^5^ cells/well were seeded in 12-well plates. After 24 h, the cells were washed twice with 1 mL phosphate-buffered saline (PBS) and incubated with FITC-labeled peptides (5 µM) in BEGM for 30 min at 37 °C. The cells were then washed three times with ice-cold PBS to remove cell surface-bound peptides. After trypsinization, the cells were collected by centrifugation (1000 × *g* for 5 min), washed and finally resuspended in 1 mL ice-cold PBS. The internalized FITC was measured using fluorescence-activated cell sorting on a FACSCalibur flow cytometer (Becton Dickinson, San Jose, CA, USA) at emission and excitation wavelengths of 510 and 530 nm, respectively. Assays were performed in triplicate and repeated at least three times. Flow cytometric analyses were accomplished using WinMDI v.2.8 software.

### Preparation of insulin/TCTP-PTD analog mixtures

To evaluate the efficacy of TCTP-PTD analogs as carriers for insulin delivery via the nasal route, insulin/PTD mixtures were prepared by simple mixing. Briefly, 10 mg insulin powder was dissolved in 1 mL 10 mM HCl. The solution was diluted in 10 mM phosphate buffer (pH 6.4) and normalized with 10 mM NaOH to obtain a final insulin concentration of 1.16 mg/mL. The TCTP-PTD analogs were also dissolved in 10 mM phosphate buffer (pH 6.4). For intranasal application, equal volumes of insulin (0.1 mM final concentration) and the TCTP-PTD analog (final concentration, 0.1 or 0.25 mM) were mixed by gentle pipetting. Each mixture was visually inspected to confirm turbidity due to precipitation. Not all insulin/PTD mixtures were clear. The turbidity of the mixtures was measured as optical density (OD), as previously reported (Landreh et al., 2012). Briefly, 50 µL 0.2 mM insulin in 10 mM phosphate buffer (pH 6.4) was dispensed into the well of a 96-well microtiter plate (Nunc, Rochester, NY). An equal volume of 0.2 or 0.5 mM PTD dissolved in the same buffer was added, and the absorbance at 500 nm measured using a microplate spectrophotometer.

### Pharmacokinetics of insulin/TCTP-PTD analog mixtures in rats

Rats were fasted for 16 h with access to water, anesthetized by intraperitoneal injection of sodium pentobarbital (60 mg/kg), and placed in the supine position. To evaluate nasal insulin absorption, the insulin/PTD solution (an insulin dose of 1 IU/kg) was administered into the right nostril of the rats, using a pipette. As a control, insulin solution without PTD was administered (5 IU/kg). Insulin solution was also administered by subcutaneous (s.c.) route (0.25 IU/kg) to calculate the relative bioavailability (BA). Blood samples (100 µL) were collected from the rat tail 5, 10, 20, 30, 60, 90, 120 and 180 min after dosing and centrifuged at 5000 × *g* for 25 min to obtain plasma. The plasma insulin concentrations were determined using enzyme-linked immunosorbent assay (ELISA) with a commercial kit (Mercodia, Uppsala, Sweden) according to the manufacturer’s protocol.

The relative BA values of insulin administered by nasal and s.c. routes were compared. Peak plasma insulin concentration (*C*_max_) and time to reach *C*_max_ (*T*_max_) were directly determined from the plasma concentration-time curves. The area under the plasma concentration-time curve (AUC) from 0–180 min (AUC_0-180 min_) was calculated according to the trapezoid rule. Relative BA was calculated using the following formula:

$$BA(\%)=({AUC}_{nasal}\times {Dose}_{s.c.})/({AUC}_{s.c}\times {Dose}_{nasal})\times 100\%$$

### Pharmacodynamics of insulin/TCTP-PTD analog mixtures in diabetic rats

To evaluate the hypoglycemic effect of the intranasally administered insulin/PTD mixtures, a rat model of diabetes was established by the intraperitoneal administration of alloxan (100 mg/kg) dissolved in 10 mM sodium citrate buffer (pH 3.2). After 5 days, rats with fasting blood glucose levels in the range of 230–300 mg/dL were selected for the pharmacodynamic studies. To evaluate the effects of insulin on blood glucose level, rats with alloxan-induced diabetes were fasted overnight (14 h) with access to water. Then, they were anesthetized by intraperitoneal injections of sodium pentobarbital (60 mg/kg). Insulin/PTD mixtures were prepared as described above and intranasally administered to anesthetized rats (2 IU/kg) with or without l-TCTP-PTD 13 or l-TCTP-PTD 13M2. Blood samples were collected from the tip of the rat tail, and blood glucose levels were directly measured using an Accu-Chek glucose meter (Roche Diagnostics, Seoul, Korea).

To calculate the relative pharmacological BA (F%), insulin solution (1 IU/kg) was administered by s.c. injection to rats with alloxan-induced diabetes. The area above the blood glucose curve (AAC) was calculated according to the trapezoid rule. The F% was calculated using the following equation:

$$F\%=({AAC}_{nasal}\times {Dose}_{s.c.})/({AAC}_{s.c.}\times {Dose}_{nasal})\times 100\%$$

### Safety/toxicity of insulin/TCTP-PTD analog mixtures

To evaluate the safety of the nasally delivered insulin/PTD mixtures, LDH activity, an indicator of general cytotoxicity, in the nasal fluid was measured. The insulin/PTD mixtures prepared as described above were applied to the nostrils of anesthetized rats (insulin dose, 1 IU/kg). Untreated rats served as negative controls. For the positive control, rats were nasally administered 5% (w/v) sodium taurodeoxycholate. After 15 min, the nasal cavity was flushed out with 1 mL PBS. LDH activity in the wash solution was measured using a CytoTox-96 assay kit (Promega, Madison, WI, USA) according to the manufacturer’s protocol. LDH leakage into the nasal fluid after the nasal administration of 5% (w/v) sodium taurodeoxycholate was defined as 100% leakage, and LDH release from untreated rats was considered as 0% leakage.

To evaluate the toxicity of l-TCTP-PTD 13M2, a 10-day repeated-dose toxicity study was carried out using male ICR mice. Briefly, mice were randomly divided into three groups that were intraperitoneally injected with 0.9% NaCl (control group) or 1 or 10 mg/kg l-TCTP-PTD 13M2 (groups 2 and 3, respectively). Body weights of the animals were measured daily throughout the experiment. After 10 days, the mice were euthanized, and the collected blood was centrifuged for 25 min at 5000 × *g* to obtain the plasma. Blood urea nitrogen (BUN), creatinine (CRE), aspartate aminotransferase (AST) and alanine aminotransferase (ALT) levels in the plasma were determined using a biochemical analyzer (AU680; Beckman Coulter, Tokyo, Japan).

### Statistical analysis

Data were analyzed using Prism 5 software (GraphPad Inc., La Jolla, CA). Group means were compared using the Student’s *t*-test or one-way analysis of variance (ANOVA) followed by Newman–Keuls multiple comparison test. Error bars were expressed as the mean ± standard error of the mean (SEM). Statistical significance was accepted at *p* < .05.

## Results and discussion

### Design, synthesis and cellular uptake ofTCTP-PTD analogs

l-TCTP-PTD 13 was recently found to be a useful vehicle for the delivery of insulin through the nasal membrane (Bae & Lee, 2013). In this study, we investigated whether replacing residues in l-TCTP-PTD 13 could further increase the efficiency of drug delivery via the nasal route.

**Figure 1.**
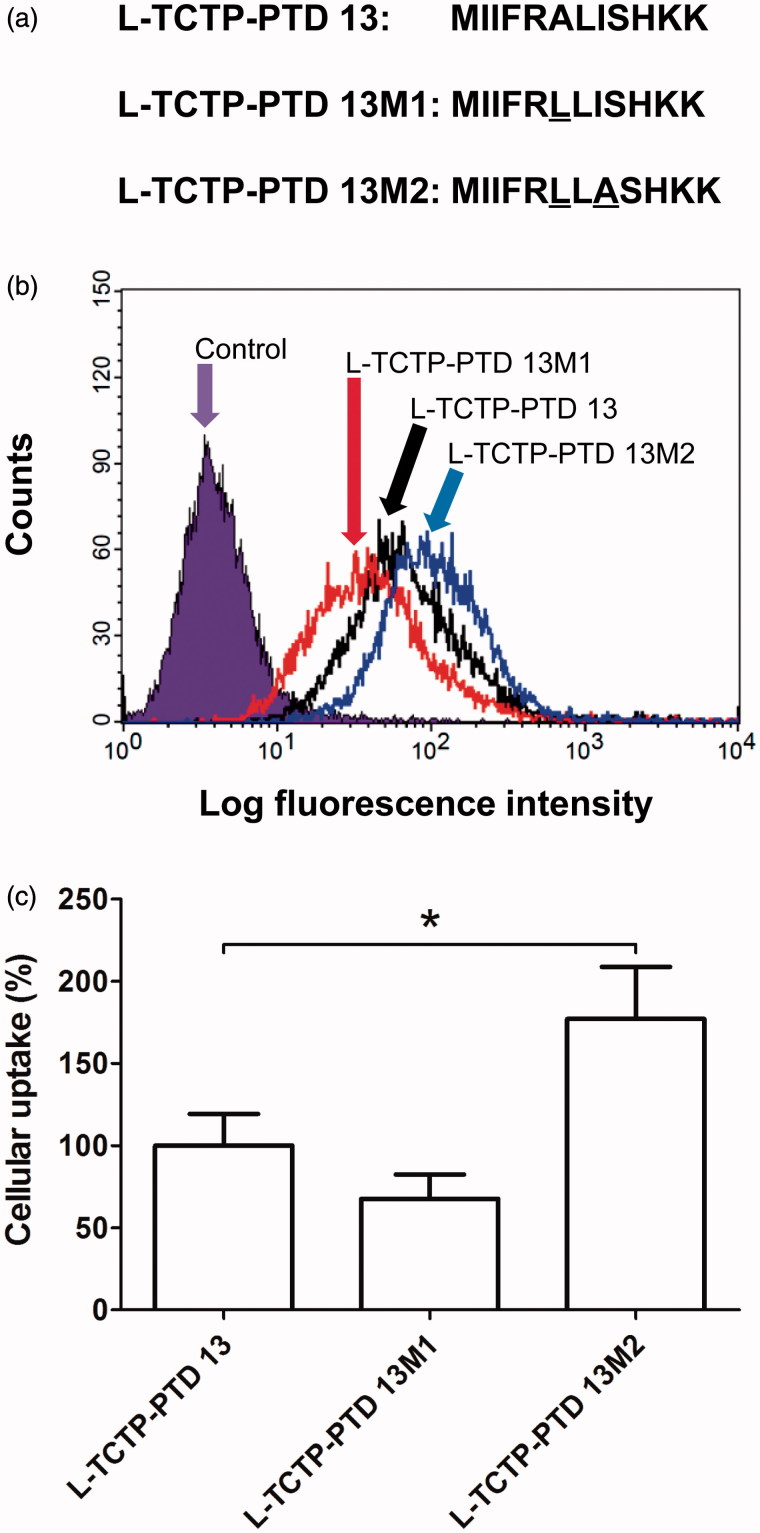
(a) Amino acid sequence of l-TCTP-PTD 13 and its analogs. Amino acid modifications in l-TCTP-PTD 13 are underlined. (b) Cellular uptake of FITC-labeled peptides in BEAS-2B cells analyzed by a flow cytometer. (c) Histograms of l-TCTP-PTD 13, l-TCTP-PTD 13M1, and l-TCTP-PTD 13M2. Each bar represents the standard deviation of three independent replicates. The mean fluorescence intensity of FITC-labeled l-TCTP-PTD 13 in BEAS-2B cells was set to 100%. *p < .05 versus FITC-labeled l-TCTP-PTD 13.

In attempts to increase the absorption of insulin delivered intranasally, we substituted residues at position 6-ALI-8 of l-TCTP-PTD 13. We did not alter the 1-MIIFR-5 and 9-SHKK-12 residues of l-TCTP-PTD 13 to preserve the capacity for translocation through the cell membrane as well as the water solubility of the peptide (Figure 1(a)) (Kim et al., 2011b). Additionally, previous studies have shown that a TCTP-PTD analog, l-TCTP-PTD 8, amino acids 1-MIIYRIAASHKK-12 with positions 6-IAA-8 did not significantly enhance the nasal insulin absorption. Therefore, it appeared that substitution of the amino acids at position 6-ALI-8 of l-TCTP-PTD 13 might improve the effectiveness of PTD as a carrier for nasal delivery of co-administered insulin.

After designing and synthesizing the TCTP-PTD-derived peptides, cellular uptake studies were performed to determine whether amino acid replacement would affect translocation capacity. We performed flow cytometry to compare the cellular uptake of fluorescent-labeled PTDs with that of the corresponding l-TCTP-PTD 13 by BEAS-2B cells. Based on the amount of fluorescent-labeled PTDs in the cells, we concluded that after replacement of one or more amino acids of the peptides, the peptides still retained their cell-penetrating ability (Figure 1(b,c)). We also found that the l-TCTP-PTD 13M2 exhibited 1.77-fold higher uptake than l-TCTP-PTD 13 in the BEAS-2B cells, whereas the capacity of l-TCTP-PTD 13M1 was lower (Figure 1(c), *p* < .05). Our previous study confirmed that the overall hydrophobicity of L-TCTP-PTD 13 was essential for the cellular uptake activity, taking into account the results of alanine substitution analysis of each residue of wild-type TCTP-PTD and biochemical properties such as peptide charge, isoelectric point and polarity (Kim et al. 2011b). Thus, we replaced the 6th residue A with more hydrophobic residue, L at the 6-ALI-8 position to increase the overall hydrophobicity of L-TCTP-PTD 13. However, the cellular uptake efficacy of L-TCTP-PTD 13M1 was not improved compared with L-TCTP-PTD 13. When the residues 6-LLI-8 are introduced in L-TCTP-PTD 13M1, the combination of hydrophobic amino acid residues containing a relatively large side chain may cause steric hindrance, resulting in a change in overall structural conformation and a decrease in solubility. Continuously, the double substitution (A6L and I8A) in l-TCTP-PTD 13 was attempted to reduce the steric hindrance without significantly affecting the overall hydrophobicity. The efficacy of l-TCTP-PTD 13M2 was significantly enhanced.

### Pharmacokinetic studies in normal rats

**Figure 2.**
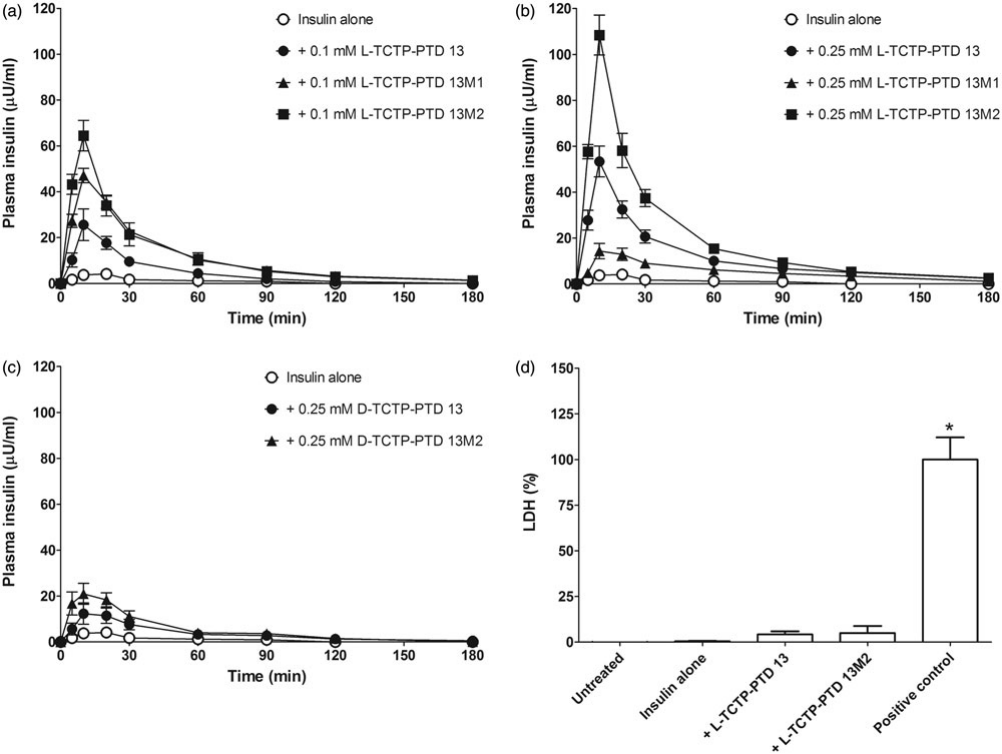
(a and b) Plasma insulin concentration in normal rats following intranasal administration of insulin in the presence of 0.1 mM (a) or 0.25 mM (b) TCTP-PTD analogs containing l-amino acids. (c) Plasma insulin concentration following intranasal administration of insulin with 0.25 mM d-TCTP-PTD analogs. Insulin doses were 5 and 1 IU/kg for insulin alone and insulin plus PTD, respectively. Vertical bars indicate means ± SEM (*n* = 5–7). (d) LDH leakage in nasal fluid of normal rats following intranasal administration of insulin (1 IU/kg) with different PTDs. Sodium taurodeoxycholate (5% w/v) served as a positive control. Each bar represents mean ± SEM (*n* = 6). *p < .01 versus insulin alone.

To evaluate the ability of TCTP-PTD analogs to enhance insulin permeation through the nasal epithelium, we performed pharmacokinetic studies in normal rats. We first evaluated the absorption-enhancing properties of intranasally administered insulin plus l-form TCTP-PTD analog formulations. PTD concentrations were selected based on previous studies (Bae & Lee, 2013) and our preliminary experiments (data not shown) in which we determined the optimal molar ratio of insulin to PTD that would enhance nasal insulin absorption. Insulin (1 IU/kg) administered alone intranasally was scarcely detectable in the plasma of rats; a dose more than five times higher was required to achieve measurable plasma insulin levels. The presence of 0.1 or 0.25 mM of the l-TCTP-PTD analog markedly increased plasma insulin levels following intranasal administration at 1 IU/kg (Figure 2(a,b)). Table 1 summarizes the pharmacokinetic parameters of the insulin concentration-time profiles. We discovered that l-TCTP-PTD 13M2 more effectively enhanced insulin absorption than l-TCTP-PTD 13 did. The relative insulin BA values of insulin alone, plus 0.25 mM l-TCTP-PTD 13, and plus 0.25 mM l-TCTP-PTD 13M2 were 0.4%, 22.1%, and 37.1% respectively, which was 1.68 times higher in the presence of 0.25 mM l-TCTP PTD 13M2 than in the presence of 0.25 mM l-TCTP-PTD 13 (*p* < .05).

**Table 1.** Pharmacokinetic parameters following intranasal administration of insulin plus TCTP-PTD analogs in normal rats.

Formulation	Dose (IU/kg)	*T*_max_ (min)	*C*_max_ (μU/mL)	AUC (μU/mL·min)	BA (%)
Insulin alone	5	16.0 ± 2.4	4.9 ± 0.9	180.5 ± 37.6	0.4 ± 0.1
+0.1 mM l-TCTP-PTD 13	1	12.0 ± 2.0	26.4 ± 7.8	847.1 ± 132.9	9.0 ± 1.4
+0.1 mM l-TCTP-PTD 13M1	1	10.0 ± 0.0	47.1 ± 3.1	1966.3 ± 130.5	20.9 ± 1.4
+0.1 mM l-TCTP-PTD 13M2	1	10.0 ± 0.0	64.5 ± 4.0	2121.9 ± 306.2	22.5 ± 3.2
+0.25 mM l-TCTP-PTD 13	1	10.0 ± 0.0	53.4 ± 6.7	2081.7 ± 238.6	22.1 ± 2.5
+0.25 mM l-TCTP-PTD 13M1	1	13.3 ± 2.1	15.0 ± 3.3	951.7 ± 225.9	10.1 ± 2.4
+0.25 mM l-TCTP-PTD 13M2	1	10.0 ± 0.0	108.5 ± 8.7	3494.4 ± 284.7	37.1 ± 3.0
+0.25 mM d-TCTP-PTD 13	1	14.0 ± 2.4	13.8 ± 4.2	645.5 ± 191.2	6.8 ± 2.0
+0.25 mM d-TCTP-PTD 13M2	1	13.0 ± 3.0	23.0 ± 5.0	949.7 ± 243.6	10.1 ± 2.6
Insulin (s.c.)	0.25	14 .0 ± 4.8	40.9 ± 0.9	2356.4 ± 106.2	100

Values are expressed as means ± SEM (*n* = 5–7).

AUC: area under the curve; BA: relative bioavailability compared to s.c.; *C*_max_: maximum concentration; s.c.: subcutaneous; *T*_max_: time to reach maximum concentration (*C*_max_).

Furthermore, l-TCTP-PTD 13 and l-TCTP-PTD 13M2 but not l-TCTP-PTD 13M1 showed dose-dependent effects of PTD on nasal insulin delivery (Figure 2(a,b)). We found that the insulin/l-TCTP-PTD 13M1 mixture showed a high degree of turbidity and precipitation (Supplementary Figure S1). This phenomenon has also been observed for l-penetratin and its analogs in other studies, which found that increased PTD-insulin molar ratio increased the turbidity, indicating the formation of larger complexes (Kristensen et al. 2015b). Consequently, the increased insolubility and low dissolution rate could decrease the ability of PTD to transport insulin across the epithelial layer of nasal mucosa. While insulin plus a higher concentration of l-TCTP-PTD 13M1 (0.25 mM) had a negative effect on nasal absorption of insulin, a lower concentration of l-TCTP-PTD 13M1 (0.1 mM) significantly increased the BA compared with that of insulin plus 0.1 mM l-TCTP-PTD 13 (*p* < .05). Although insulin solution also becomes turbid in the presence of a 0.1 mM l-TCTP-PTD 13M1 solution, the physicochemical properties of the insulin/PTD complex could favor the transport of insulin through the nasal membrane. Thus, substituting position 6 in l-TCTP-PTD 13 with leucine rather than alanine residues enhanced nasal absorption of insulin considerably.

The l-TCTP-PTD 13M1 and l-TCTP-PTD 13M2 combinations differ by a single amino acid at position 8, and substituting alanine at this position not only decreased the precipitation of the insoluble complexes but also increased the efficiency of nasal insulin absorption. Among the l-form TCTP-PTD analogs, l-TCTP-PTD 13M2 was the most efficient carrier for intranasal insulin delivery. Therefore, l-TCTP-PTD 13M2 was further evaluated to determine the effect of d-amino acids in PTDs on nasal insulin absorption and toxicity.

In a previous study, we showed that changing the amino acid stereochemistry of TCTP-PTD 13 from the l- to the d-form enhanced the absorption of an antigen (ovalbumin) through the nasal mucosa (Bae et al., 2016). We speculated that improving d-TCTP-PTD 13 stability would promote intranasal delivery of the antigen. To investigate this possibility, we evaluated whether d-TCTP-PTD analogs function as insulin carriers. Although nasal absorption of insulin was increased in the presence of d-TCTP-PTD analogs, neither d-TCTP-PTD 13 nor d-TCTP-PTD 13M2 was superior to their l-form counterparts (Figure 2(c)). The relative BA values of insulin plus 0.25 mM d-TCTP-PTD 13 or 0.25 mM d-TCTP-PTD 13M2 were 6.8% and 10.1%, respectively (Table 1).

Turbidity measurements indicated that adding d-TCTP-PTD analogs to insulin solution was more prone to inducing a high degree of complex formation than adding their l-amino acid counterparts (Supplementary Figure S2). Therefore, the d-TCTP-PTD analogs could negatively affect insulin delivery through the nasal mucosa. Since d-TCTP-PTD 13 was a more potent antigen carrier than l-TCTP-PTD 13, it seems that the nasal absorption rate depends on the cargo. Similarly, a previous study reported that insulin plus l-penetratin was more effective than d-penetratin, which showed a negligible insulin absorption following intranasal administration of insulin (Khafagy et al., 2009). This lower absorption was likely due to a lower rate of insulin release from the insulin/d-penetratin complex in nasal enzyme fluid than from the insulin/l-penetratin complex (Khafagy et al., 2009).

Our pharmacokinetic studies indicated that amino acid sequence modification of TCTP-PTD improved intranasal drug delivery. However, how the amino acid composition of l-TCTP-PTD 13M2 increases insulin delivery via the nasal route relative to l-TCTP-PTD 13 remains unclear. As shown in Figure 1(b,c), we found a significant cellular internalization of l-TCTP-PTD 13M2 compared to that of l-TCTP-PTD 13. This may suggest enhanced permeation of the carrier peptide-insulin complex through the nasal mucosa. The binding of the bioactive cargo to PTD and their mixing ratio have been proposed to be important factors affecting nasal drug delivery (Khafagy et al., 2010; Bae & Lee, 2013). An analysis of the intermolecular association using a surface plasmon resonance-based binding assay showed little difference in the binding characteristics of l-TCTP-PTD 13 and l-TCTP-PTD 13M2 (data not shown). The intermolecular interactions between insulin and carrier peptides might be affected by the different components of nasal cavity secretions. Thus, elucidation of the role of insulin and PTD binding characteristics in enhancing nasal insulin absorption may require further detailed analysis of the association and dissociation rates of PTD-insulin interactions under the conditions of the mucus layer.

### LDH activity in nasal fluid

We investigated whether enhanced nasal delivery of insulin is accompanied by histological alterations to the nasal membrane following co-administration with l-TCTP-PTD 13M2. LDH activity was evaluated as a marker of nasal membrane damage. Insulin plus l-TCTP-PTD 13M2 did not alter LDH activity in rats treated with either insulin alone or insulin plus l-TCTP-PTD 13 (Figure 2(d)). In contrast, the positive control sodium taurodeoxycholate increased LDH leakage compared to insulin alone. These results indicate that l-TCTP-PTD 13M2 enhanced nasal delivery of insulin without causing damage to nasal mucous membranes.

### Pharmacological studies in diabetic rats

**Figure 3.**
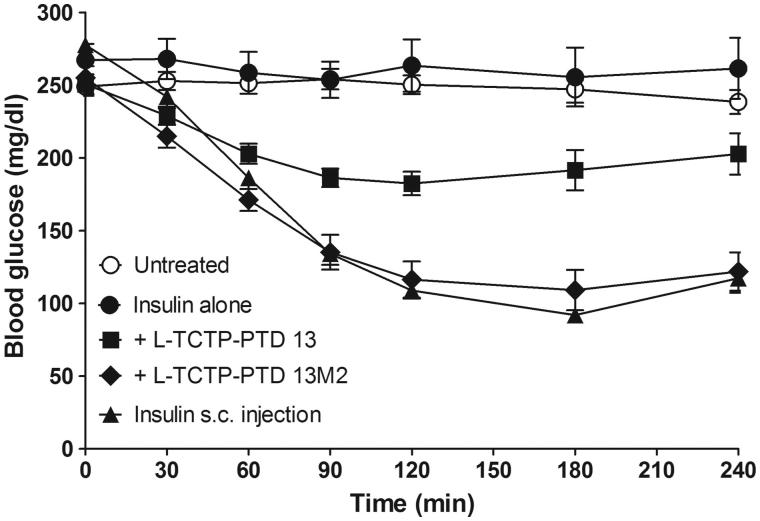
Changes in blood glucose levels in rats with alloxan-induced diabetes following intranasal administration of insulin plus l-TCTP-PTD analogs. Insulin doses were 2 and 1 IU/kg for administration by the nasal route and s.c. injection, respectively. Vertical bars indicate means ± SEM (*n* = 6–8).

To confirm whether PTD-delivered insulin has therapeutic effects, we carried out pharmacological studies to evaluate nasal insulin absorption and its hypoglycemic effect in a rat model of alloxan-induced diabetes. No hypoglycemic effect was observed relative to untreated rats (negative control) following the intranasal administration of 1 IU/kg insulin alone. Therefore, we used an insulin dose of 2 IU/kg to induce a measurable hypoglycemic effect. The hypoglycemic effect of 1 IU/kg insulin delivered by s.c. injection was also assessed to calculate the relative F%. Blood glucose levels after intranasal administration of insulin alone were similar to those in untreated rats (Figure 3).

As expected based on previous findings, blood glucose levels were markedly reduced in diabetic rats treated with insulin plus l-TCTP-PTD analogs compared with levels in rats treated with insulin alone. The minimum values attained were ∼73% and ∼45% of the initial level at 120 and 180 min for insulin plus l-TCTP-PTD 13 and insulin plus l-TCTP-PTD 13M2, respectively. The relative F% of insulin plus l-TCTP-PTD 13M2 was 42.3%, which was significantly higher than the value of 22.4% for insulin plus l-TCTP-PTD 13 (*p* < .05, Table 2). These results demonstrate that intranasal administration of insulin plus l-TCTP-PTD 13M2 induces a significant hypoglycemic effect in diabetic rats.

**Table 2.** Pharmacodynamics of insulin in rats with alloxan-induced diabetes.

Formulation	Dose (IU/kg)	AAC_0–240 min_ (% glucose/min)	F%
Insulin alone	2	721.0 ± 478.2	3.2 ± 2.1
+0.25 mM l-TCTP-PTD 13	2	4860.1 ± 424.5	22.4 ± 1.7
+0.25 mM l-TCTP-PTD 13M2	2	9604.5 ± 1204.6	42.3 ± 5.3
Insulin (s.c.)	1	11354.3 ± 507.8	100

Values are expressed as means ± SEM (*n* = 6–8).

AAC: area above the blood glucose curve; F%: relative pharmacological bioavailability compared to s.c.; s.c.: subcutaneous.

It has been reported that both PTD-fused recombinant protein and conjugation of a PTD to a cargo can improve the efficiency of cargo delivery; however, these approaches can reduce the biological effects of the cargo (Kristensen et al. 2015a). Co-administration of a cargo and PTD is preferable for its simplicity, the automatic release of cargo in live cells or tissues, and preservation of cargo function. Although we have demonstrated that the newly designed TCTP-PTD analog is an efficient trans-nasal peptide carrier of insulin, a major limitation to its clinical application is PTD-induced aggregate formation. It has also been reported that the disadvantage of the mixing method for complexation of PTD with insulin for intestinal delivery is a reduction in insulin solubility, which decreases the efficiency of PTD-mediated insulin delivery (Kamei et al., 2008; He et al., 2013). Thus, the solubility problems of PTD-based insulin delivery need to be resolved to accomplish successful intranasal delivery.

#### Safety of l-TCTP-PTD 13M2

**Figure 4.**
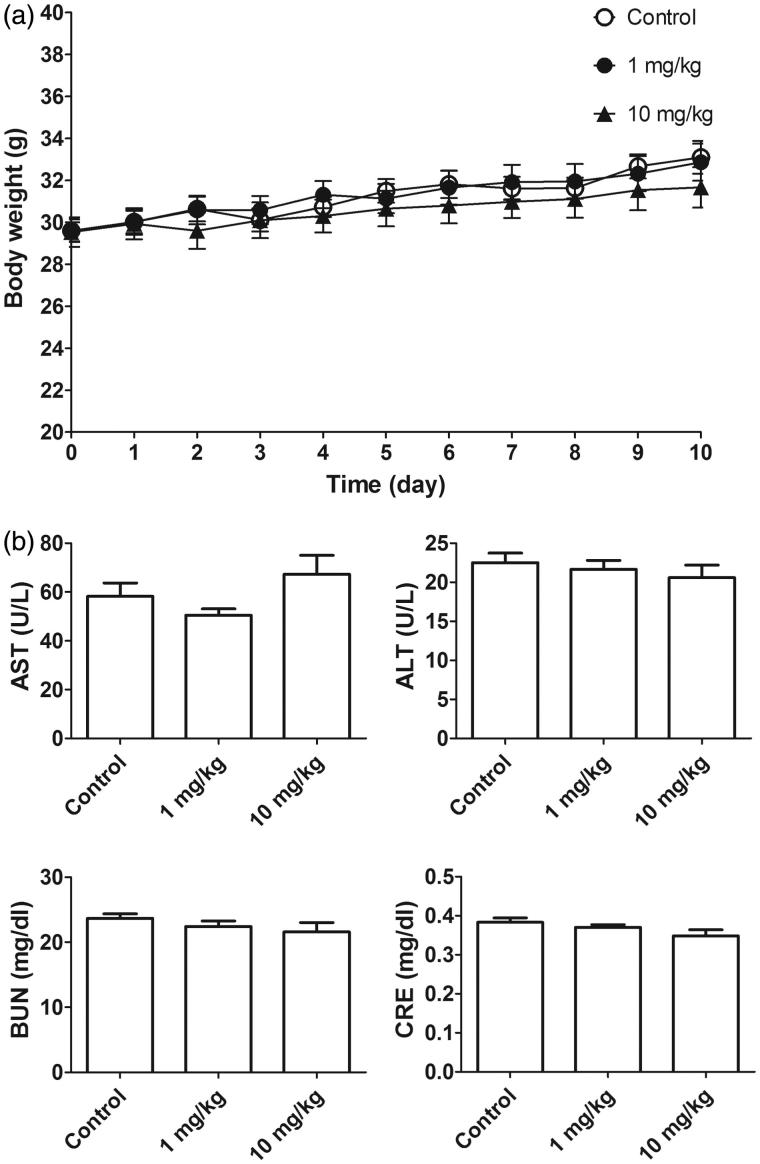
(a) Body weight of normal mice (*n* = 6) after daily intraperitoneal injection of l-TCTPPTD 13M2 for 10 days. (b) Biochemical analysis of AST, ALT, BUN, and CRE levels.

In our previous study, we found that l-TCTP-PTD 13 can penetrate the nasal membrane to enter the circulation. Accordingly, l-TCTP-PTD 13M2 may penetrate the nasal mucosa and cause undesirable side effects. We used normal mice for toxicity studies and monitored body weight gain as an indirect indicator of general good health. We also measured the plasma levels of AST, ALT, BUN, and CRE in l-TCTP-PTD 13M2-injected mice. The liver transaminases (AST and ALT) served as biomarkers of liver injury while BUN and CRE were used as markers of kidney function associated with kidney pathologies. Mice were administered daily doses of l-TCTP-PTD 13M2 at 1 or 10 mg·kg^−1^·Day^−1^ for 10 days. There was no significant difference in body weight (Figure 4(a)) or AST, ALT, BUN, and CRE levels (Figure 4(b)) between the PTD-treated and control groups indicating that the newly designed peptide caused no detectable toxicity.

## Conclusions

In conclusion, we have developed novel peptide carriers to enhance nasal drug delivery. Furthermore, we have shown that l-TCTP-PTD 13M2 (MIIFRLLASHKK) effectively promoted the nasal absorption of insulin and improved the relative BA of insulin without damaging nasal tissues. Additional studies are clearly necessary to clarify the detailed mechanism by which PTD improves nasal drug absorption, but our findings suggest that new TCTP-PTD analogs can be designed for effective nasal drug delivery.
